# Factors Associated With a High Motivation to Undergo Fertility Preservation in Female Cancer Patients

**DOI:** 10.3389/fpsyg.2021.782073

**Published:** 2021-12-16

**Authors:** Valentina Elisabetta Di Mattei, Gaia Perego, Paola Taranto, Paola M. V. Rancoita, Mariangela Maglione, Lisa Notarianni, Giorgia Mangili, Alice Bergamini, Raffaella Cioffi, Enrico Papaleo, Massimo Candiani

**Affiliations:** ^1^School of Psychology, Vita-Salute San Raffaele University, Milan, Italy; ^2^Clinical and Health Psychology Unit, IRCCS San Raffaele Scientific Institute, Milan, Italy; ^3^Department of Psychology, University of Milano-Bicocca, Milan, Italy; ^4^University Centre for Statistics in the Biomedical Sciences (CUSSB), Vita-Salute San Raffaele University, Milan, Italy; ^5^School of Medicine, Vita-Salute San Raffaele University, Milan, Italy; ^6^Obstetrics and Gynecology Unit, IRCCS San Raffaele Scientific Institute, Milan, Italy

**Keywords:** cryopreservation, fertility, oncology, motivation, counseling, psychology

## Abstract

**Objective:** Fertility loss due to cancer treatment can be a devastating experience for women and the couple. Undergoing fertility preservation can be a complex decision from both a medical and emotional point of view. The aim of the present study was to evaluate which socio-demographic and psychological factors predict a high motivation to undergo fertility preservation.

**Methods:** Fifty-eight female cancer patients who accessed an Oncofertility Unit completed: a questionnaire to collect socio-demographic characteristics and the level of motivation, the Beck-Depression Inventory-II, the State-Trait Anxiety Inventory-Y, and the Fertility Problem Inventory.

**Results:** Almost half of the sample (44.8%) declared a high motivation. At multiple logistic regression analysis only the “Need for parenthood” subscale of the FPI predicted a high motivation. We alternatively evaluated as possible predictor the construct “Representations about the importance of parenthood” (i.e., the sum of the “Need for Parenthood” and “Rejection of childfree lifestyle” subscales) in place of the two separate subscales. At multiple logistic regression analysis, only this variable predicted a high motivation to undergo fertility preservation.

**Conclusion:** The most important predictor of a high motivation to undergo fertility preservation is the individual desire for parenthood. This implies that, regardless of socio-demographic characteristics, any woman of childbearing age should receive an appropriate counseling about fertility preservation.

## Introduction

Advances in cancer diagnosis and treatment have led to the increase of patients’ survival rates, consequently bringing more attention to the long-term side effects of cancer treatments and their impact on quality of life. Among these, gonadotoxicity is considered the principal cause of infertility in young women with cancer (Rodriguez-Wallberg and Oktay, 2014; Anderson et al., 2015).

More than 9.2 million women have been diagnosed with cancer in 2020 (GLOBOCAN, 2020), with about 10% diagnosed during reproductive age (Ward et al., 2014; Siegel et al., 2016). About 75% of women aged 18–45 years with a cancer diagnosis are interested in the possibility of having children (Letourneau et al., 2012a; Deshpande et al., 2015), but this desire could be unfulfilled. The risk of infertility, due to the disease itself or to gonadotoxic treatments, depends on different factors such as the age of the patient, the type of therapy, as well as the tumor site, stage, and grade (Lee et al., 2006). Fertility loss due to cancer treatment can be a devastating experience for young women who may not have satisfied their need for parenthood. Infertility is a significant concern for cancer patients at diagnosis (Partridge et al., 2004), who may experience higher levels of anxiety and depressive symptoms than non-cancer-related infertile women (Lawson et al., 2014, 2015). Furthermore, many cancer patients continue to report heightened reproductive concerns during survivorship, which has been linked to poorer mental and emotional health outcomes (Gorman et al., 2010, 2015; Armuand et al., 2014; Ussher and Perz, 2018, 2019). In fact, for some women, the failure of the motherhood project is far more painful than the cancer itself (La Rosa et al., 2020). Moreover, both female and male cancer patients seem to experience fertility-related psychological distress from diagnosis to survival (Logan and Anazodo, 2019; Logan et al., 2019). Furthermore, fertility loss does not only affect the individual, but may have a dyadic impact. In fact, the literature on cancer survivors shows that potential infertility is associated with feelings of pressure on the relationship, fights, frustration, concerns, or breakups (Lehmann et al., 2018, 2019). In addition, for some of them, not being able to have children was experienced as not being able to “provide” within a relationship (Lehmann et al., 2018, 2019). Moreover, a study on the reaction of female cancer patients to the risk of fertility loss reported the presence of doubts about the possibility of remarrying and feelings of regret about what the husband was also witnessing in their couple. A combination of emotional reactions of fear, anxiety, worry, loneliness, guilt, sorrow, depression, and regret also emerged (Hasanpoor-Azghdy et al., 2014).

Currently, new procedures that allow fertility preservation before cancer treatments are available, such as oocyte or ovarian tissue cryopreservation. In order to better address fertility-related concerns, professional societies such as the American Society of Clinical Oncology have published international guidelines indicating that all young adults and children with cancer should receive timely fertility counseling before treatment to safeguard their reproductive potential (Oktay et al., 2018).

Oncofertility (fertility and oncological care) thus rose as a new interdisciplinary field, which includes fertility preservation discussion and management, the management of sexual dysfunction, hormonal dysfunction, complex contraception, and psychosocial support (Anazodo et al., 2018). It involves the cooperation of different health professionals, such as gynecologic oncologists, reproductive medicine gynecologists, biologists, general oncologists, endocrinologists and psychologists, with the common objective to provide counseling about fertility preservation options for cancer patients (Lange et al., 2013; Sigismondi et al., 2015).

Despite best practice recommendations, there are many barriers in providing oncofertility services (Quinn et al., 2008). A systematic review indicates that most cancer patients in reproductive age (i.e., 14–45 years) do not receive adequate information about fertility risks and preservation strategies before initiating cancer treatment (Logan et al., 2018). The barriers to fertility preservation are multifactorial: limited time for clinical encounters, focus on treatment planning, reluctance to discuss sensitive subjects such as sexuality and fertility, poor knowledge about fertility preservation techniques (Quinn et al., 2009), distress and anxiety of the patient, and financial problems (Quinn et al., 2008).

However, several studies have underlined that receiving specialized counseling about cancer-related infertility and fertility preservation is associated with less regret and greater quality of life in cancer survivors (Letourneau et al., 2012a; Laganà et al., 2017). In fact, the decision to undergo fertility preservation before cancer treatments is not an easy one to make: patients may feel overwhelmed, adding further concerns to the understandable worry about their lives and their future as a result of the recent diagnosis. Moreover, because these techniques can be undertaken only before starting cancer treatment, these decisions usually have to be taken rapidly (Mangili et al., 2017). The literature shows that fertility preservation counseling is associated with better decision-making outcomes in the long term, specifically less regret and conflict about the decision, better coping with the burden of cancer treatment (Letourneau et al., 2012a; Mersereau et al., 2013; Benedict et al., 2015; Deshpande et al., 2015), and improved social well-being (Skaczkowski et al., 2018). For these reasons, oncofertility care should always be an integral part of cancer care, allowing for informed patient counseling and decision-making and reducing long-term impact on mental health and quality of life.

Currently, the concept of a shared decision-making process, which allows for the provision of all necessary information to the patient in order to guarantee an optimal quality of life, is gaining support. Including patients’ psychological point of view allows them to consider future projects that may occur after the disease and thus endure long-term planning. Since patients are generally referred to oncofertility specialists on an emergency basis, they frequently have limited information, many fears and questions, and varying expectations about the therapeutic process. Consistently, the psychological management of this path is critical, as the psychologist can welcome and support patients before their oncofertility visit.

Few studies in the available literature have investigated which variables could intervene in the decision-making process about undergoing fertility preservation techniques in cancer patients. Some of these studies have shown that this decision may be influenced by personal factors (i.e., age, gender, relationship status, having children, and personal beliefs), illness factors (i.e., prognosis, stage of cancer, and time to commence oncological treatment) and fertility preservation-related factors (i.e., expected burden, duration, and costs of treatments) (Logan and Anazodo, 2019; Melo et al., 2019). However, very few studies have investigated which psychological factors may influence the choice to undergo fertility preservation techniques in cancer patients (Hershberger et al., 2016; Melo et al., 2019), highlighting the role of a high desire for motherhood and a strong individual value for the possibility of guaranteeing a future pregnancy (Hershberger et al., 2016; Melo et al., 2019).

The aim of the present study is thus to investigate which sociodemographic and psychological variables predict a high motivation to undergo fertility preservation, first of all, evaluating the sociodemographic and psychological characteristics of female cancer patients that are referred to the Oncofertility Unit of the San Raffaele Hospital before gonadotoxic treatment. Considering that the San Raffaele Hospital is an Italian reference center for fertility preservation, we are not able to intercept female cancer patients who decline such possibility and all the patients who come to the oncofertility examination have usually already been informed by their oncologists about the effects of therapies on fertility and about fertility preservation opportunities. Therefore, they are usually already motivated to undergo fertility preservation techniques. As such, the hypothesis that the present study addressed is that women who decide to undergo fertility preservation may have specific adaptive psychological characteristics that allow them to cope with the disease in the short-term and to preserve the mental space for future planning and thus for possible parenthood.

## Materials and Methods

### Sample Selection and Recruitment

Female patients referred to the San Raffaele Hospital Oncofertility Unit after cancer diagnosis and before gonadotoxic treatment between March 2016 and June 2020 were invited to participate in the study. All patients received oncologic and fertility counseling from a group of specialists including a gynecologic oncologist, a reproductive gynecologist and a psychologist.

Eligible women had to meet the following criteria: being at least 18 years old, having been recently diagnosed with cancer, speaking and understanding Italian, having at least an elementary school certificate, and agreeing to voluntarily participate in the research. Following these criteria, 58 women took part in the research.

The study was carried out following the guidelines of the San Raffaele Hospital Ethics Committee (protocol N. 149/INT/2019), in accordance with the Declaration of Helsinki. A psychologist illustrated the research objectives during the psychological counseling which precedes the gynecologic examination and obtained written informed consent from every participant.

### Measures

A psychologist collected the sociodemographic characteristics (age, relationship status, number of children, education, number of abortions) of the patients. Motivation to undergo fertility preservation was measured on an 10-point Likert scale (1 = not at all; 10 = very much). Participants were then asked to complete self-report questionnaires measuring psychological symptoms and infertility-related stress.

Psychological scales were selected according to the few studies that investigated which factors influence the decision to preserve fertility and identified aspects related to the individual desire for a future pregnancy (Baysal et al., 2015; Hershberger et al., 2016; Flink et al., 2017a; Melo et al., 2019). Moreover, we focused on anxiety and depression because they are generally associated with difficulties in decision-making (Bishop and Gagne, 2018; Köther et al., 2021).

The Beck Depression Inventory-II (BDI-II–Beck et al., 1996) is a self-report questionnaire designed to evaluate depressive symptoms. This questionnaire is composed of 21 items that ask respondents to rate the severity of their depressive symptoms during the past 2 weeks. Responses are given on a 4-point Likert scale (from 0 to 3). Individual item scores are summed to create a total severity score ranging from 0 to 63; different severity levels have been defined on an empirical basis (Dozois et al., 1998): minimum depression (scores of 0–13); mild depression (scores of 14–19); moderate depression (scores of 20–28); severe depression (scores of 29–63). The BDI-II has good psychometric properties, with Cronbach’s alpha coefficient ranging from 0.92 for outpatient samples to 0.93 for non-clinical samples (Beck et al., 1996). The Italian validation study confirmed its good psychometric properties, with internal consistency values ranging between 0.80 and 0.86 and test–retest reliability of 0.76 (Ghisi et al., 2006).

The State-Trait Anxiety Inventory-Y (STAI-Y–Spielberger et al., 1983) is a widely used self-report measure of state and trait anxiety. It is composed of two subscales, which include 20 items each: the state subscale measures anxiety related to a specific situation or time-period (at the moment of questionnaire completion), while the trait subscale measures relatively stable anxiety (how an individual feels on a day-to-day basis). Responses are given on a 4-point Likert scale (from 1 to 4) with total scores ranging from 20 to 80 for both the state and trait subscale. Scores are grouped into three categories (Elliott, 1993): low anxiety (scores of 20–39), medium anxiety (scores of 40–59), and high anxiety (scores of 60–80). The STAI-Y scales have good reliability, with Cronbach’s alpha coefficient between 0.83 and 0.95 (Spielberger et al., 1983). The Italian version of the STAI-Y showed internal consistency values ranging between 0.91 and 0.95 for the state subscale and between 0.85 and 0.90 for the trait subscale; test–retest reliability is 0.49 for the state subscale and 0.82 for the trait subscale (Pedrabissi and Santinello, 1989).

The Fertility Problem Inventory (FPI–Newton et al., 1999) is a self-administered questionnaire that measures infertility-related stress. The instrument comprises 46 items organized in five subscales (“Social concern,” “Sexual concern,” “Relational concern,” “Need for parenthood,” and “Rejection of child-free lifestyle”), with answers given on a 6-point Likert scale (from 1 “strongly disagree” to 6 “strongly agree”). A global measure of stress is also derivable by summing the scores of all five subscales. The FPI has good discriminant and convergent validity. In addition, all scales show good reliability, as indicated by Cronbach’s alpha reliability coefficients between 0.77 and 0.93 (Newton et al., 1999).

The Italian validation study of the questionnaire (Donarelli et al., 2015) showed that the scales defined in the original version have adequate internal consistency, with alpha reliability coefficient values between 0.71 and 0.93 for all subscales, excluding the “Rejection of child-free lifestyle” subscale (α = 0.66). Moreover, both Moura-Ramos et al. (2012) and Donarelli et al. (2015) found a strong correlation (*r* = 0.55 and *r* = 0.64, respectively), between the two subscales “Need for Parenthood” and “Rejection of child-free lifestyle.” In particular, Moura-Ramos et al. (2012) proposed a bifactorial model of the FPI, including the “Impact on Life Domains” (i.e., “Social concern,” “Sexual concern,” and “Relational concern”) and the “Representations about the Importance of Parenthood” (i.e., “Need for parenthood” and “Rejection of child-free lifestyle”) dimensions. Therefore, we used the Italian version of the FPI (Donarelli et al., 2015), considering the original version’s factors of Newton et al. (1999), but we also considered in the analysis the larger construct “Representations about the importance of parenthood” (including the two subscales “Need for parenthood” and “Rejection of child-free lifestyle”) as defined in Moura-Ramos et al. (2012).

### Statistical Analysis

Comparisons of the distribution of the psychometric scales with respect to values in the reference populations were performed with the Wilcoxon test. The comparison was made with values of the reference populations for the BDI-II (Ghisi et al., 2006) and the STAI-Y (Pedrabissi and Santinello, 1989), and with the population of infertile women for the FPI and the STAI-state subscale (Donarelli et al., 2015). Bonferroni’s correction was applied to account for multiple testing. Spearman’s correlation coefficient was computed for evaluating the correlation between psychometric scales.

Multiple logistic regression analysis was used to evaluate the possible predictors of a high motivation (i.e., motivation = 10) to undergo cryopreservation. The covariates considered in the analysis were: sociodemographic variables, state and trait anxiety, the level of depression and either the FPI subscales “Need for parenthood” and “Rejection of a childfree lifestyle” or the FPI construct “Representation about the importance of parenthood.” Each final model was obtained with a backward variable selection.

The level of significance was set at 0.05. All statistical analyses were performed using IBM SPSS Statistics version 20 and R version 3.5.0.

## Results

Descriptive statistics for sociodemographic variables are reported in Table 1. The sample consisted of 58 women recently diagnosed with cancer, aged between 20 and 42 years (median = 33; IQR = 28, 75–37); the majority of them had a partner (82.8%, *n* = 48) and had no children (86.2%, *n* = 50). In addition, the majority of the sample had never had an abortion in the past (82.8%, *n* = 48) and 53.4% (*n* = 31) had at least a bachelor’s degree.

**TABLE 1:** Descriptive statistics of socio-demographic variables.

Variable	N	*n*%
Children	58	
*Yes*		8 (13.8%)
*No*		50 (86.2%)
In a relationship	58	
*Yes*		48 (82.8%)
*No*		10 (17.2%)
Bachelor’s degree	58	
*Yes*		31 (53.4%)
*No*		27 (46.6%)
Previous abortions	58	
*Yes*		10 (17.2%)
*No*		48 (82.8%)

Table 2 shows descriptive statistics for the psychometric variables, together with the results of the comparisons with reference values from the general population and the population of infertile women. The level of depression in our sample resulted to be significantly higher than that of the general population (adj. *p* = 0.018), with a distribution of the scores (median = 9; IQR = 6.00–14.25) that indicates mainly a minimum level of depression. Trait anxiety was lower than that of the general population (adj. *p* = 0.007), with a distribution (median = 38; IQR = 31.00–44.00) indicating low anxiety for most of the patients, and state anxiety was lower than that of the population of infertile women (*p* = 0.003), with a median value (median = 43; IQR = 35.00–55.00) indicating medium anxiety. There was no significant difference in state anxiety levels when compared to the general population (adj. *p* = 0.124). As regards the FPI, we observed a significant difference between our sample and the population of infertile women only for the subscale “Rejection of childfree lifestyle” (adj. *p* < 0.001): our sample reported significantly lower scores than that of the population of infertile women.

**TABLE 2:** Descriptive statistics of psychometric variables and comparison with reference values through Wilcoxon test with *p*-values adjusted with Bonferroni’s correction.

Variable	N	Median	IQR	Reference value	*p*-value	Adjusted *p*-value
BDI-II	58	9	6.00–14.25	7.29	0.002	0.018*
STAI trait	58	38	31.00–44.00	42.06	0.001	0.007**
STAI state	58	43	35.00–55.00	39.62	0.012	0.124
				52.11^a^	<0.001	0.003**
FPI social concern	53	19	15.00–25.00	21.82^a^	0.150	1.000
FPI sexual concern	53	14	10.00–18.00	16.39^a^	0.017	0.167
FPI relationship concern	51	21	16.00–31.00	22.39^a^	0.415	1.000
FPI need for parenthood	58	40	30.25–47.00	40.97^a^	0.108	1.000
FPI rejection of a childfree lifestyle	58	26.50	20.25–32.00	32.64^a^	<0.001	<0.001**
FPI global stress	48	124.50	97.75–142.00	134.2^a^	0.010	0.099
FPI representations about the importance of parenthood	58	64	52,00–77,25	–	–	–

*^a^Reference value for the population of infertile women.*

*p < 0.05 and **p < 0.01.

*BDI-II, Beck Depression Inventory-II; STAI, State-Trait Anxiety Inventory; FPI, Fertility Problem Inventory.*

As expected from the design of the study, the patients in the sample declared mostly high levels of motivation to undergo fertility preservation treatments (median = 9, IQR = 8–10, min = 3, max = 10). Thus, in the study, a high motivation was defined as equal to 10. Almost half of the sample (44.8%; *n* = 26) declared a high motivation. Multiple logistic regression analysis was performed in order to identify which factors predicted a high motivation to undergo fertility preservation techniques. In the analysis, all collected variables were considered except for the FPI subscales “Social concern,” “Sexual concern,” and “Relational concern.” For the FPI subscales, we only included the “Need for parenthood” and “Rejection of childfree lifestyle” subscales because they reflect the individual investment in parenthood. After backward variable selection, only the “Need for parenthood” subscale of the FPI was retained in the model; the higher the values of the subscale, the higher the probability to have a high motivation (OR = 1,114 [1.040–1.194], *p* = 0,002). In the literature a moderate correlation was observed between the “Need for parenthood” and the “Rejection of childfree lifestyle” subscales (Moura-Ramos et al., 2012; Donarelli et al., 2015), which was confirmed also in our sample (rho = 0.682; *p* < 0.001), suggesting the possibility of a partial overlap between the meanings of the two subscales. Thus, we alternatively evaluated as possible predictor the construct “Representations about the importance of parenthood” (defined as the sum of the two subscales by Moura-Ramos et al., 2012) in place of the two separate subscales. At multiple logistic regression analysis, only this variable was retained in the final model with a similar role (OR = 1.061 [1.021–1.104], *p* = 0.003).

## Discussion

The choice to preserve fertility before antineoplastic treatments can be a complex process both from a medical and emotional point of view; consequently, not all cancer patients may have the mental space to evaluate this possibility (Di Mattei et al., 2020).

Our findings show that our sample display significantly higher levels of depression as compared to the general population; this finding could be explained considering that these patients have recently received a life-threatening diagnosis. However, their scores can be classified as minimum depression (Dozois et al., 1998); it is possible that women with higher levels of depression do not come to the Oncofertility Unit, as they may consider it an additional burden.

As compared to the general population, our sample also shows lower levels of trait anxiety, which can be interpreted as low anxiety (Elliott, 1993). This personality trait may promote the possibility to undertake further procedures other than cancer treatment. Our patients also show medium state anxiety levels (Elliott, 1993), probably as a reaction to their disease. However, these do not significantly differ from the reference values of the general population. As compared to the population of infertile women, the state anxiety levels in our sample are significantly lower. These could be explained by the fact that infertile women have overt reproductive difficulties, and they are actually seeking pregnancy when their anxiety levels are measured.

As regards the subscales and the global scale of the FPI, we observed no significant difference between our sample and the population of infertile women, except for the “Rejection of childfree lifestyle” subscale, whose scores in our sample are significantly lower than those of the population of infertile women. This shows that cancer patients and infertile women both experience infertility as a stressful condition: infertility seems to represent an event of great impact for every woman on a social, relational and/or individual level. Furthermore, we can hypothesize that the significantly lower scores obtained in the “Rejection of childfree lifestyle” subscale in women with cancer could be explained considering that infertile women are usually evaluated while they are trying to have a child; therefore, it is plausible that they have a more negative view of a life without children, while cancer patients undergo fertility preservation techniques with the purpose of a future investment, and not for imminent motherhood.

As far as we know, few studies have investigated which psychological factors may influence the choice to undergo fertility preservation in cancer patients, and even fewer studies have investigated the level of motivation. The purpose of this study was therefore to evaluate which sociodemographic and psychological variables predict a high motivation to undergo fertility preservation in female cancer patients.

Our results show that sociodemographic variables such as age, relationship status, number of children, education and number of abortions do not significantly predict a high motivation to undergo fertility treatment. The literature includes mixed findings. In line with our results, Flink et al. (2017a) highlighted that age, relationship status, education and having children are not significantly associated with adherence to fertility preservation techniques. Similarly, the same authors (Flink et al., 2017b) reported no significant association between sociodemographic characteristics, such as relationship status or having children, and the complex process of decision-making about fertility preservation in cancer patients, while highlighting a small influence of factors such as cultural beliefs and social pressure. Also Hershberger et al. (2016) found no significant difference in age, relationship status, having children, and education between a group of cancer patients that decided to preserve fertility and another group that rejected this option.

However, other studies report different findings. For example, Letourneau et al. (2012a) found that women with cancer who do not have college degree, are over 35 years old, and have children are less likely to access to fertility preservation. Other studies highlight a significant association between not having children and a greater probability of proceeding with fertility preservation techniques (von Wolff et al., 2016; Melo et al., 2019). A possible interpretation is that women with certain sociodemographic characteristics are less likely to receive adequate oncofertility counseling. It can be hypothesized that women with cancer who already have children prioritize antineoplastic treatments over fertility preservation; however, another possibility is that oncologists are more likely to refer women without children to fertility preservation specialists.

The discrepancy with our findings may be related to the different objective of these studies, which focus on the actual decision rather than the level of motivation to undergo fertility preservation techniques. Although not having children can influence the decision to undergo fertility preservation, this factor may not be so decisive in affecting the level of motivation: other factors may have a greater impact on the motivation to preserve fertility. Specifically, we found that the only two factors predicting a high motivation to undergo fertility preservation were the “Need for parenthood” subscale and the “Representations about the importance of parenthood” construct. Thus, we can hypothesize that the strong desire for motherhood as an essential life value can predict a high motivation to preserve fertility. This is in line with other studies, showing that the desire for motherhood and the importance of parenthood are crucial factors influencing the choice to preserve fertility before cancer treatment (Baysal et al., 2015; Hershberger et al., 2016; Flink et al., 2017a), while women who decline this path place more emphasis on surviving cancer (Hershberger et al., 2016). Similarly, Melo et al. (2019) found that another predictor of the decision to preserve fertility is the possibility to have a child in the future.

Finally, depression and anxiety levels did not significantly predict a high motivation. Although no studies in the literature have investigated this aspect, it could be assumed that the levels found within our sample are not clinically relevant; this could allow patients to decide to access the service and preserve fertility, but could be irrelevant for a high motivation, which remains closely associated to the importance of parenthood.

### Limitations and Future Directions

Some limitations of the present research must be acknowledged. First, this is a preliminary single-center study conducted on a relatively small sample. Second, the research was conducted on patients who are referred by their oncologists to the Oncofertility Unit, both inside the San Raffaele Hospital and from other cancer centers; this implies that the women who participated in the study have high levels of motivation from the start.

Future studies could collect data on larger samples that are more representative of the population of cancer patients who decide to undergo to fertility preservation techniques; it would also be desirable to recruit patients who refuse the option of preserving fertility in order to identify which factors influence the choice itself. Furthermore, it could be interesting to extend the analysis to male cancer patients, in order to investigate possible gender differences. Finally, including an evaluation of patients’ partners would be useful to understand couples’ needs in such a challenging situation.

### Clinical Implications and Conclusion

The results of our study support the initial hypothesis, highlighting that women who decide to undergo fertility preservation show psychological characteristics, such as low levels of depression and anxiety, that may favor the possibility to preserve the mental space for future planning and thus for possible parenthood.

The crucial clinical implication underlined by our results is that the most important predictor of a high motivation to undergo fertility preservation is the strong desire for parenthood, regardless of the sociodemographic characteristics which could potentially limit the referral of patients to fertility specialists. In fact, several studies report that patients with certain socio-demographic characteristics (e.g., older age, single status, lower education, and already having children at the time of diagnosis) are less likely to be referred to fertility preservation specialists (Goodman et al., 2012; Letourneau et al., 2012b; Bastings et al., 2014; Chin et al., 2016; Lawson et al., 2017). Conversely, our findings highlight the importance of giving every woman the chance to be informed about fertility preservation options, thereby promoting a shared decision-making process, and fostering a better long-term quality of life (Letourneau et al., 2012a). Consistently, we recommend addressing this issue both at the time of diagnosis, to allow for timely intervention, and after treatment, to re-evaluate the available options (Loscalzo and Clark, 2007), possibly including multidisciplinary professionals.

The present findings are particularly relevant also in a dyadic perspective, given the literature indicating that the risk of infertility due to cancer treatment can add a further challenge in the early stages of diagnosis and treatment, and can determine important reactions for patients such as depression, anxiety, distress, worry, and a negative impact on sexual well-being, quality of life, and couple relationship (Lawson et al., 2014; Perz et al., 2014; Lehmann et al., 2018; Ussher and Perz, 2019). Considering that individual and couple needs and desires may differ depending on life projects, circumstances, and socio-cultural context (Jones and Hunter, 1996; Butler and Green, 1998), the possibility to be informed and consciously choose about fertility preservation can have a positive effect on both the individual and the couple’s quality of life.

## Data Availability Statement

The datasets presented in this article are not readily available because data cannot be shared as participants did not provide written informed consent for it. Requests should be directed to GP, g.perego23@campus.unimib.it.

## Ethics Statement

The studies involving human participants were reviewed and approved by the Ethics Committee of the San Raffaele Hospital. The patients/participants provided their written informed consent to participate in this study.

## Author Contributions

VEDM, GP, and PT contributed to the conception and design of the study. PT, PR, MM, and LN organized the database. PR and LN performed the statistical analysis. GP, PT, PR, and MM wrote the first draft of the manuscript. VEDM, GM, AB, RC, EP, and MC revised the manuscript critically. All authors read and approved the final version.

## Conflict of Interest

The authors declare that the research was conducted in the absence of any commercial or financial relationships that could be construed as a potential conflict of interest.

## Publisher’s Note

All claims expressed in this article are solely those of the authors and do not necessarily represent those of their affiliated organizations, or those of the publisher, the editors and the reviewers. Any product that may be evaluated in this article, or claim that may be made by its manufacturer, is not guaranteed or endorsed by the publisher.
